# SMTrackR: an R/Bioconductor package for mapping protein binding at individual DNA molecules

**DOI:** 10.1093/bioadv/vbag091

**Published:** 2026-05-15

**Authors:** Aashna Bansal, Himani Barmola, Shivam Yadav, Satyanarayan Rao

**Affiliations:** Department of Biosciences and Bioengineering, Indian Institute of Technology Roorkee, Uttarakhand, 247667, India; Department of Biosciences and Bioengineering, Indian Institute of Technology Roorkee, Uttarakhand, 247667, India; Department of Biosciences and Bioengineering, Indian Institute of Technology Roorkee, Uttarakhand, 247667, India; Department of Biosciences and Bioengineering, Indian Institute of Technology Roorkee, Uttarakhand, 247667, India

## Abstract

**Motivation:**

Single-molecule assays like NOMe-seq, dSMF, and Nanopore are superior to DNase-seq and ATAC-seq as they do not destroy DNA. Thus, they enable quantification of all three, that is, protein-free, Transcription Factor-bound, and histone-complex-bound states. But a user-friendly tool to visualize and quantify such states is lacking. Here, we present SMTrackR, an R/Bioconductor package to visualize protein-DNA binding states on individual sequenced DNA molecules.

**Results:**

SMTrackR queries the single-molecule footprint database we built and hosted at Galaxy Server. It comprises BigBed files generated from NOMe-seq, dSMF, and Nanopore (SMAC-seq) datasets. SMTrackR exploits UCSC REST API to query a BigBed file and plot footprint heatmap categorized in different binding states, as well as report their occupancies. Additionally, this package generates a Gviz-enabled script to visualize these single molecules on gene tracks.

**Availability and implementation:**

The SMTrackR tool is implemented in the statistical programming language R and is available as a Bioconductor package, SMTrackR (https://bioconductor.org/packages/3.23/bioc/html/SMTrackR.html). The GitHub repository at https://github.com/satyanarayan-rao/SMTrackR has latest updates. The installation time is less than five minutes given the dependent packages are installed. The tool is also available as a web version https://smtrackrest.iitr.ac.in/. A function is provided to use local BigBed file for users who wish to use unpublished data. A fully automated pipeline to generate such BigBed files is available at https://github.com/satyanarayan-rao/SMF_for_SMThub, and https://github.com/satyanarayan-rao/dSMF_for_SMThub.

## 1 Introduction

Transcription Factors (TFs), a special class of proteins capable of binding DNA, are at the apex of gene regulation as they are central to the precise spatiotemporal gene expression (Furlong and Levine 2018). In eukaryotes, nucleosomes present a formidable barrier to their binding (Ramachandran and Henikoff 2016). This active competition contributes to cell-type-specific gene expression profiles. From a substrate (DNA) perspective, there could be three possible states at any given binding locus, that is, (i) TF-bound, (ii) Histone-complex bound, and (iii) protein-free or unbound. Revealing all three states gives a complete picture of the binding profile. However, some of the widely used methods either TF-target specific such as ChIP-seq and CUT&RUN or target-agnostic such as DNase-seq and MNase-seq fail to capture unbound (naked DNA) state (Krebs *et al*. 2017, Skene and Henikoff 2017). But, a chemical probe (GpC methyltransferase)-based bulk assay method, pioneered by Kelly *et al*. (2012) enabled detection of all states, predominantly TFs and histone complex binding as footprints, and naked DNA as a lack of footprint at individual sequenced DNA molecules, facilitating a high-resolution map. In contrast to nuclease- or transposase-based methods, it informs all three possible states, that is, protein-free DNA, TF-bound, and histone-bound, at a given locus *in vivo*. Since then, several research groups adopted the technology and even assayed at the single-cell level to address key biological questions (Guo *et al*. 2017, Gu *et al*. 2019, Wang *et al*. 2021). On the same principle, variants like dSMF, SMAC-seq, and Fiber-seq, where different methyltransferases, whether alone or in tandem, were used to achieve about base pair footprint size resolution (Krebs *et al*. 2017, Abdulhay *et al*. 2020, Shipony *et al*. 2020, Stergachis *et al*. 2020, Altemose *et al*. 2022, Nanda *et al*. 2024). Full use of the data, however, has not been utilized to address many more questions, or at least as a useful resource. It is potentially due to a lack of appropriate databases and an interface. Here, we developed SMTrackR, a front-end R/Bioconductor package to visualize and quantify protein-DNA binding states at a locus of interest using publicly available single-molecule assay datasets. We also provide a web-server version accessible at https://smtrackrest.iitr.ac.in/. Our approach differs from existing tools, such as NOMePlot and SingleMoleculeFootprinting, primarily in two key aspects (Requena *et al*. 2019, Kleinendorst *et al*. 2021, Rao and Ramachandran 2022). First, SMTrackR not only provides visualization but also explicitly classifies individual DNA molecules into distinct protein–DNA binding states, enabling quantitative comparison of binding-state occupancies across conditions. Second, SMTrackR adopts a modular architecture where it uses the UCSC REST API to efficiently query preprocessed BigBed tracks hosted at public servers, for instance, Galaxy, thereby supporting scalable, locus-specific, rapid visualization from numerous single-molecule footprinting datasets. Also, researchers are free to use publicly accessible BigBed tracks as per their needs or use their unpublished data to generate desired visualizations. Codes to generate BigBed tracks for NOMe-seq and dSMF are available as snakemake pipelines (please see Availability and implementation).

## 2 Methods

### 2.1 SMTHub

**Figure 1:**
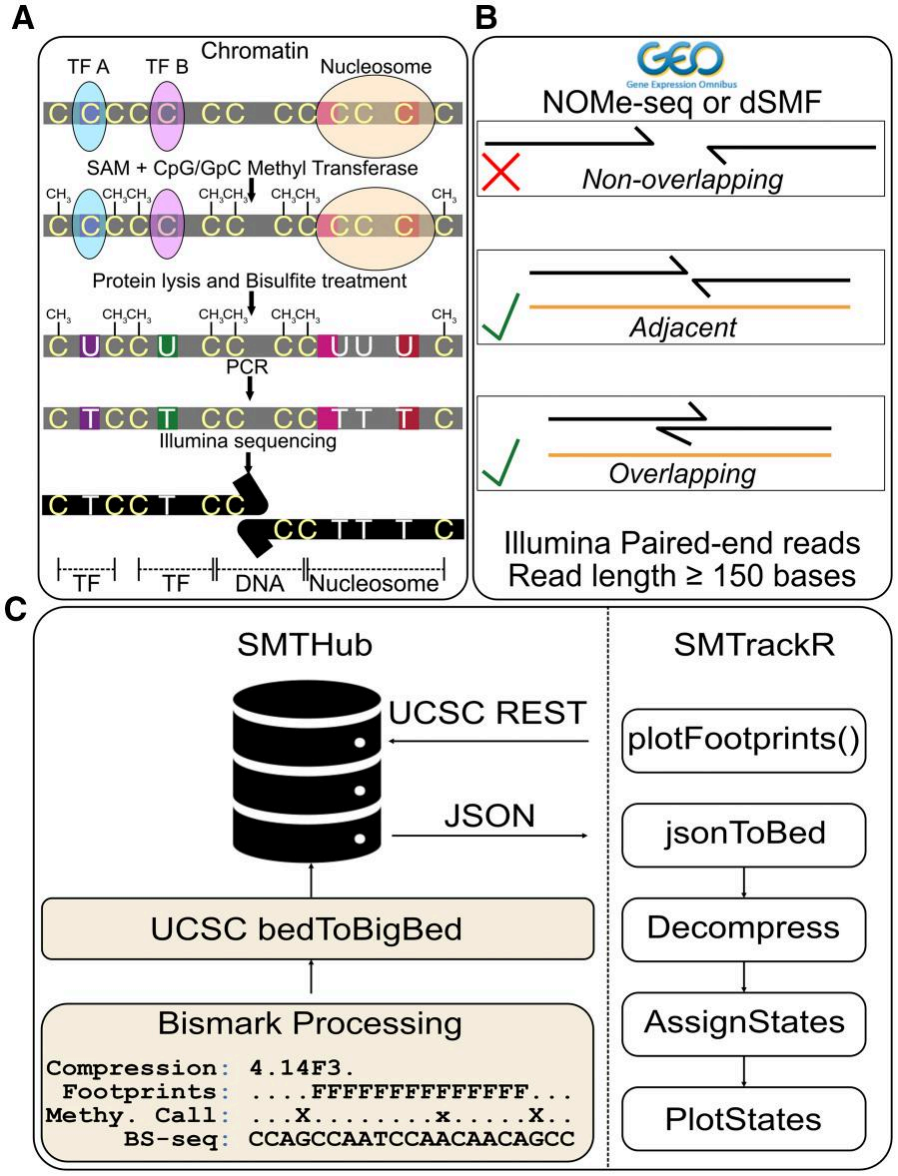
Single-molecule footprinting data processing. (A) Schematic representation of detecting binding states on a 300-base-long DNA molecule from dual Enzyme Single Molecule Footprinting (dSMF) assay. CH_3_ represents methylation of the C5 carbon of cytosine in CpG or GpC context. (B) Sequencing data selection criteria: NOMe-seq or dSMF paired-end reads with at least 150 bp length are selected. Adjacent and overlapping reads are kept for single-molecule footprinting while discarding the non-overlapping reads. (C) Bismark is used for methylation calls on individual molecules, followed by footprint calls, which are converted to BigBed and stored in SMTHub. Footprints are retrieved using SMTrackR via the UCSC REST API for visualization and analysis.

We performed massive sequence analysis on NOMe-seq and dSMF data (∼1.5 TB) from several studies to call protein-DNA binding footprints. To reliably map all three states (Fig. 1A), particularly nucleosomes, Illumina sequenced paired-end reads with greater than 150 bases, and reads either adjacent or overlapping were used as filters (Fig. 1B; Supplementary Table 1). A complete description to annotate footprints on individual sequenced DNA molecules can be found here (Rao *et al*. 2021, Rao and Ramachandran 2022). We made minor changes to adapt the NOMe-seq data to this protocol. Biological replicates were merged to generate a high-coverage footprint dataset, and, for single-cell data, pseudo-bulk footprint datasets were generated by merging the corresponding annotated cells. For example, all individual 4Cell-stage scNOMe-seq bed files with footprint information were merged to generate a single 4Cell BigBed track (Wang *et al*. 2021). When using pseudo-bulk data, we recommend users turn on the ‘remove_dup’ flag (Supplementary Fig. S1). Fully automated pipelines with a detailed description on generating bigBed from the raw paired-end fastq files are available at https://github.com/satyanarayan-rao/SMF_for_SMThub and https://github.com/satyanarayan-rao/dSMF_for_SMThub. We benchmarked one such dataset to evaluate the time and memory usage of SMTHub (Table 1).

**Table 1:** Benchmarking of SMThub.

PRJNA316148, SRR3288084 (Total Reads = 8298157)				
Ubuntu 24.04 LTS x86-64 [RAM: 32GB, Processor: Intel(R) Xeon(R) w7-2475X]				
|S|	Task type	Time (minutes)	Max Memory (GB)	Max Virtual Memory (GB)
1	Adapter Trimming	68m43s	1.00	1.00
2	Bismark Alignment	35m13s	2.00	1.95
3	Alignment to BigBed	68m43s	1.00	1.00

File sizes = 709MB (R1), 771MB (R2) (Guo *et al*. 2017).

### 2.2 SMTrackR

**Figure 2:**
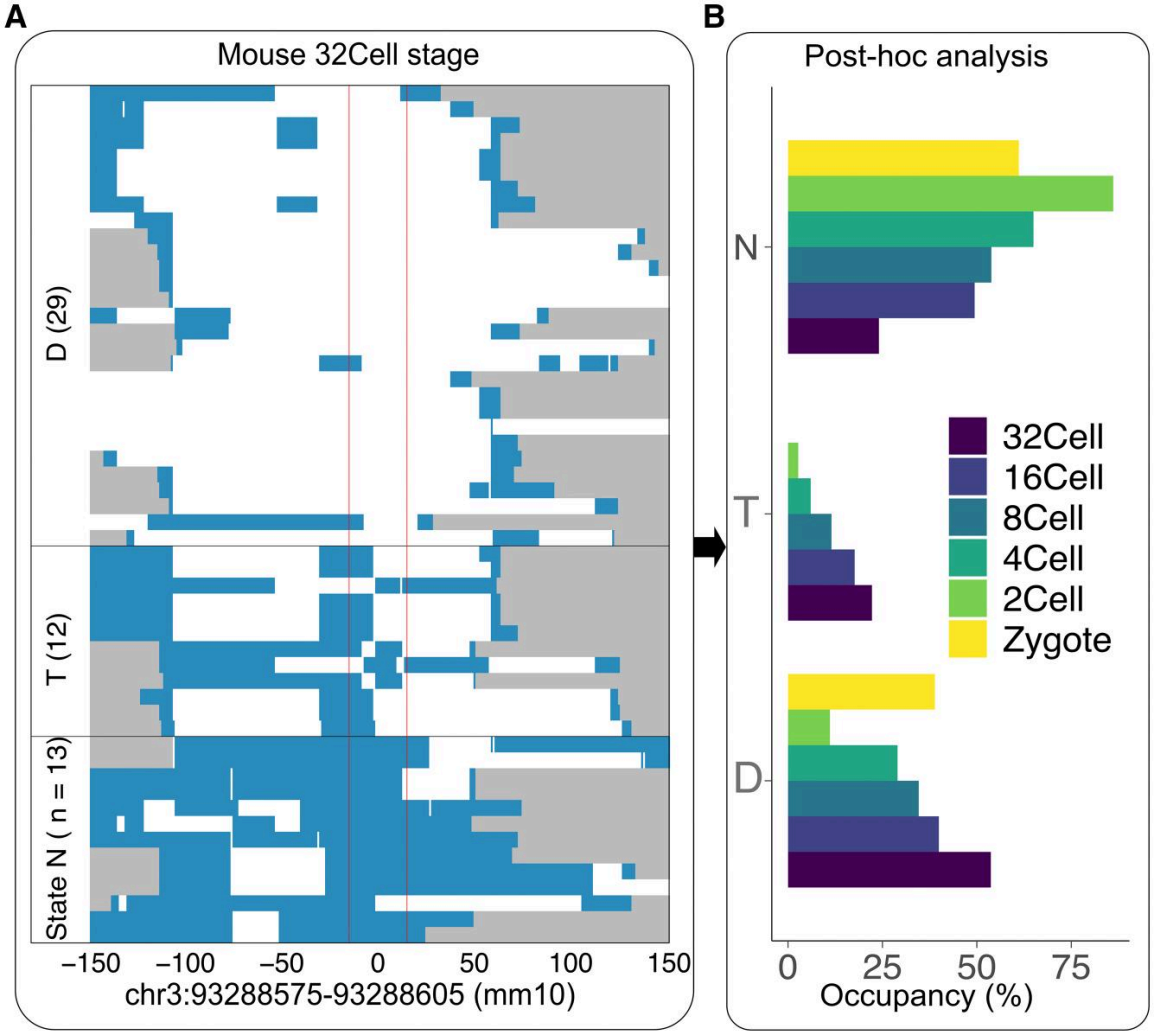
SMF resolves protein-DNA binding states. (A) Heatmap generated using SMTrackR for the theoretically derived footprint in the 32-cell stage. Molecules are grouped into three states: D (naked DNA), T (TF-bound), and N (nucleosome-bound), based on methylation patterns. Each row is a sequenced DNA molecule. White, blue, and grey colors on the DNA molecule represent naked DNA, footprint, and space fill (for molecules less than 300 bases long), respectively. (B) Analysis of dynamic changes in occupancy of the proximal region of ZGA genes using SMTrackR across different developmental stages. Nucleosome depletion increases as the mouse zygote progresses to the 32-cell stage.

SMTrackR package queries BigBed files hosted at Galaxy using USCS REST API to fetch footprint information overlapping the locus of interest (Fig. 1C). It uniquely maps BigBed files using relevant information like the organism of interest, cell-type, conditions, etc. The retrieved JSON file is then processed, which primarily involves: (i) expanding the compressed version of footprint information, and (ii) assigning binding states based on footprint length and other criteria (Rao and Ramachandran 2022). It then uses base R functions to plot the heatmap (Fig. 2A, Supplementary Fig. S2). The function also writes a TSV file with percentage occupancy, molecule counts, and other information, using which the user can do post-hoc analysis, such as probing chromatin dynamics as a function of developmental stage (Fig. 2B, Supplementary Fig. S2). The whole process is quite fast and completely depends on the number of molecules mapped to the region of interest. Benchmarking for the plotFootprints() function is shown in Supplementary Table 2. For a wider application of the package in the visualization context, we have also provided a function ′generateGvizCodeforSMF′ to integrate the heatmap with genomic annotations, such as Transcription Start Sites, exons, and more. This function generates a Gviz-compatible R script file, running which will place the heatmap under the ideogram, axis, and gene tracks (Supplementary Fig. S3) (Hahne and Ivanek 2016). Users can change the zoom levels and other parameters in the code to suit their needs.

### 2.3 Sub-genome suitable for TF footprinting with NOMe-seq

Because NOMe-seq can only utilize GpCs as probes, it has often been used to report nucleosome occupancies primarily. Interestingly, shortest *in silico* footprints, that is, distance between every first and third cytosine in the GpC context, in mammalian genome sequence alone (tested on mouse and human), we find that about 35% (37 679 878 of 107 792 112) are less than 45 base pairs long, satisfying the TF footprint size criteria (≤ 50 bp), and overlaps with regulatory element characterized by ChromHMM (Supplementary Figs. S4, S5). Thus, NOMe-seq can reliably map TF-bound states at those loci, particularly for TFs with GpC in their binding motifs. We have compiled a set of TFs (Supplementary Table 3) with such motifs and provided *in silico* footprint bed files (for human and mouse genomes) with three contiguous GpCs. A general recommendation is to consider at least 30 molecules mapped to the region of interest (see coverage calculation in Supplementary Materials).

## 3 Results

Signals from external stimuli ultimately manifest in action by TFs, activating or repressing a plethora of genes. Such scenarios lead to blocking or unblocking TF binding sites—thus, learning TF-Nucleosome dynamics is key to understanding gene regulation. SMTrackR enables this by mapping all binding states at single-molecule resolution *in vivo*. It covers deeply sequenced dSMF data from *Drosophila melanogaster* S2 cells, a widely used model cell line for numerous studies. Currently, it supports Illumina-based sequencing datasets and Oxford Nanopore Technology-based methylation calls. In the future, we plan to incorporate PacBio long reads.

Existing tools, to our knowledge, available for visualizing single-molecule methylation and chromatin accessibility include NOMePlot, SingleMoleculeFootprinting, and Methylartist (Requena *et al*. 2019, Kleinendorst *et al*. 2021, Cheetham *et al*. 2022). NOMePlot is designed to visualize Illumina-based NOMe-seq–style datasets, whereas Methylartist is primarily used for long-read methylation sequencing data from Oxford Nanopore or PacBio SMRT platforms. In contrast to the likes of SingleMoleculeFootprinting, which works with both SMF and dSMF datasets, SMTrackR enables Oxford Nanopore data visualization. The pre-processed component, SMTHub, makes this package distinct from existing tools. The REST API-based server allows rapid integration of this tool to existing cis-regulatory databases like ReMap 2022 (Hammal *et al*. 2022).

## Supplementary Material

vbag091_Supplementary_Data

## Data Availability

No experimental data was generated in this study. Publicly available single-molecule sequencing datasets were used to generate bigBed tracks. References to all used datasets are available in listTracks() function of the SMTrackR package. Users who wish to visualize data not available in the package can fill this request form: https://forms.gle/2ZHJvsxg7G1Dw49HA.
