# Supplementary material for: SMTrackR: an R/Bioconductor package for mapping protein binding at individual DNA molecules
Source: Bioinform Adv. 2026 May 15;6(1):vbag091. doi: 10.1093/bioadv/vbag091 (PMC13287992; doi:10.1093/bioadv/vbag091)
Supplement: vbag091_Supplementary_Data [file vbag091_supplementary_data.zip › supp_table2_proof_corrected.docx]

**Supplementary Table 2: Benchmarking of SMTrackR**

| ***SMTrackR::plotFootprints(); tested on R 4.5*** | | | | |
| --- | --- | --- | --- | --- |
| **Organism** | **Model** | **Locus** | **Genome Assembly** | **Time taken**  **(Seconds)** |
| *D. melanogaster* | S2 | chr2L:480290-480320 | dm6 | 16.34 |
| *D. melanogaster* | OSC | chr2L:480290-480320 | dm6 | 9.78 |
| *M. musculus* | 4Cell | chr1:171064512-171064542 | mm10 | 8.57 |
| *M. musculus* | 8Cell | chr6:87850670-87850700 | mm10 | 5.44 |
