# Supplementary material for: SMTrackR: an R/Bioconductor package for mapping protein binding at individual DNA molecules
Source: Bioinform Adv. 2026 May 15;6(1):vbag091. doi: 10.1093/bioadv/vbag091 (PMC13287992; doi:10.1093/bioadv/vbag091)
Supplement: vbag091_Supplementary_Data [file vbag091_supplementary_data.zip › 28-May-2026_012906_Supplementary_SMTrackR_proof_corrected.docx]

**Supplementary Materials**

**Determining molecule count for reliable binding state inferences**

We use the well-known coverage formula to derive the molecule count.

$$c=\frac{NL}{G}$$

Where N is the number of reads, L is the read length, and G is the effective genome size. At any locus of interest, in our case, L is about 250 (after merging paired-end Illumina reads, median length is 270), G = 180 (75 bp up- and down-stream of TF flank, 30 bp for TF). For 10x coverage, we would need about 8 molecules (180x10/250). Thus, 10 molecules should suffice to infer a state. So, a total of 30 molecules should suffice to infer all three states.

| 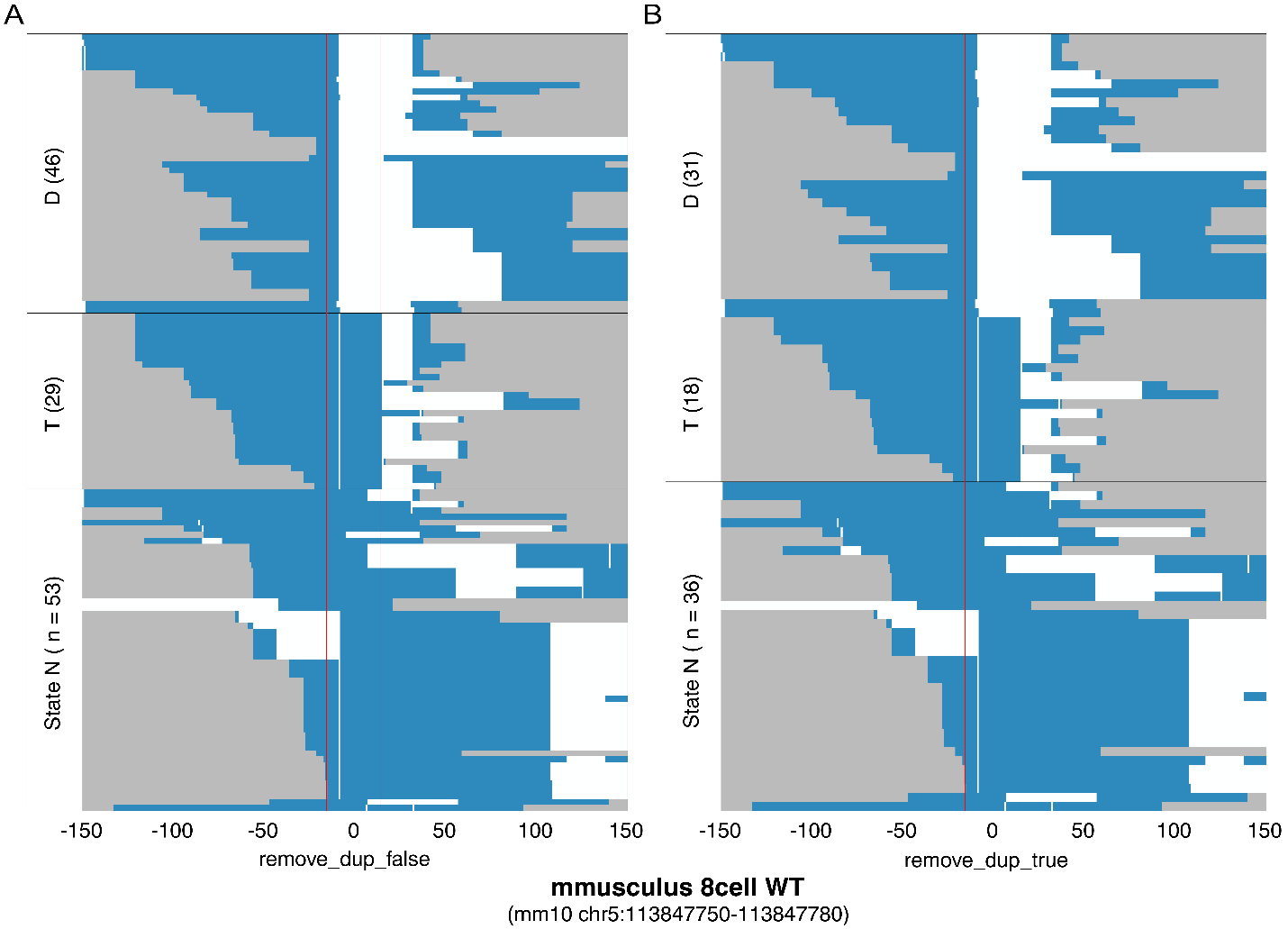 |
| --- |
| **Figure S1:** **Effect of PCR duplicates in molecule counts. (A)** generated with command: plotFootprints(organism = "mmusculus", model = "8cell", condition = "WT", genome_assembly = "mm10", type = "SMF", chromosome = "chr5", start = 113847750, end = 113847780, tr = "8cell", label = "remove_dup_true", fp_cap = 50, remove_dup = F), and **(B)** with remove_dup = T. |

| **** |
| --- |
| **Figure S2. Developmental dynamics of TF occupancy at the *cnbp* promoter. (A)** Single-molecule footprint heatmaps showing TF occupancy patterns at the *cnbp* promoter in the zygote. **(B)** Post-hoc quantitative analysis showing the percentage of molecules occupied by TFs across early embryonic stages (zygote, 2-cell, 4-cell, 8-cell, 16-cell, and 32-cell). TF occupancy is highest in the zygote and progressively decreases during early development, indicating dynamic and stage-specific regulation of the *cnbp* promoter. |

| 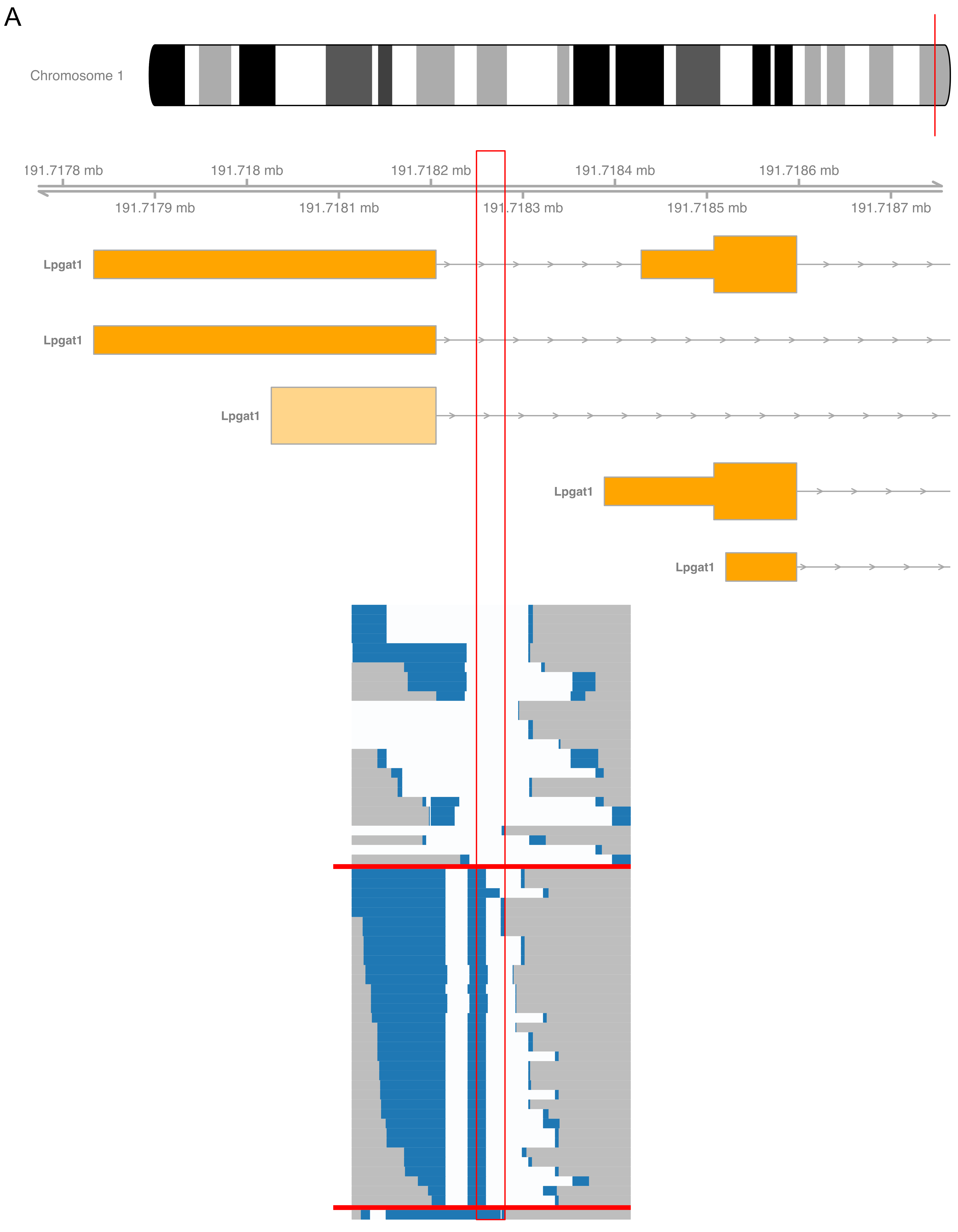 |
| --- |
| **Figure S3: (A)** plot generated using Gviz-compatible code**.** |

| 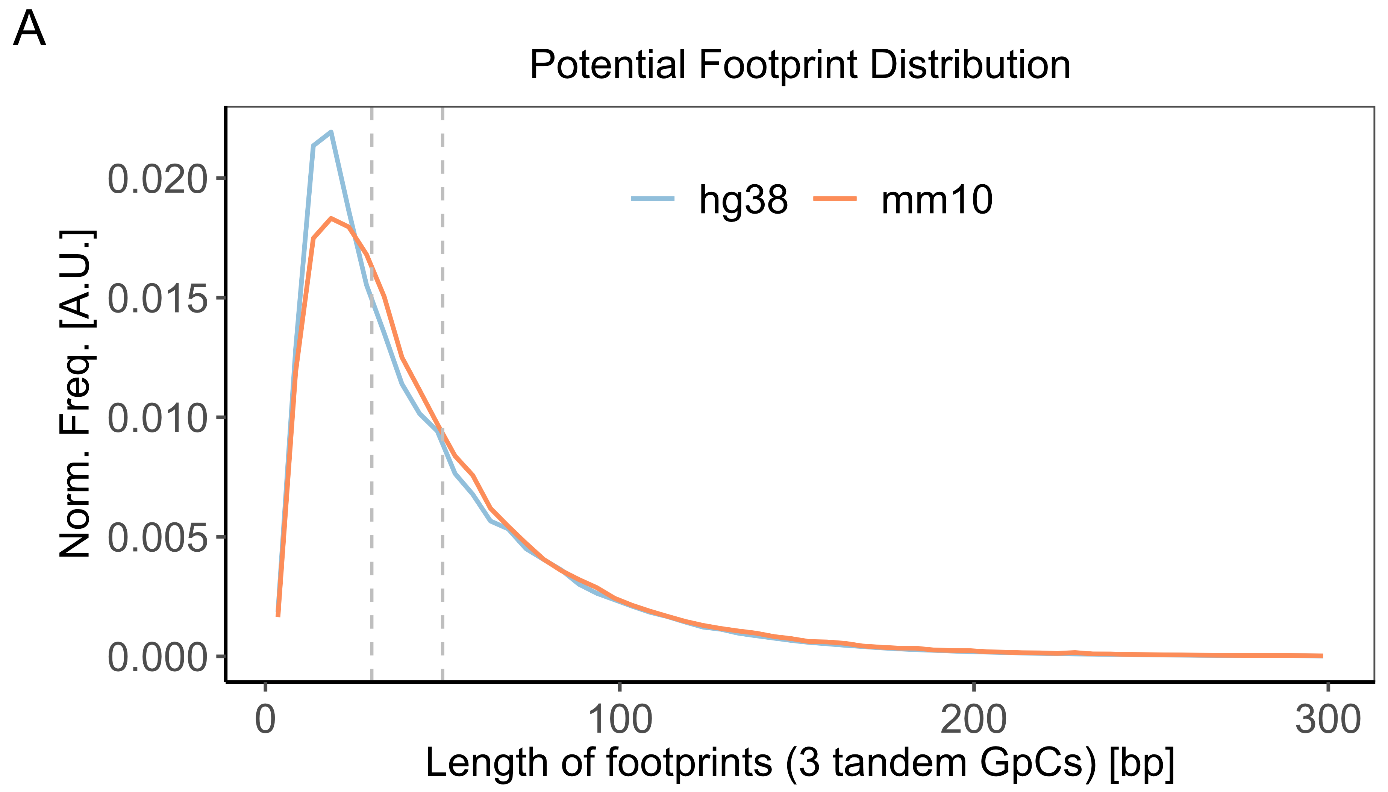 |
| --- |
| **Figure S4: Theoretical shortest footprint length distribution with GpC as probe**. **(A)** In Human and Mouse, genome-wide footprints were defined using three contiguous GpCs, potentially giving the shortest possible footprint. Vertical grey lines are at 30 and 50 base pairs (bp), respectively. |

| **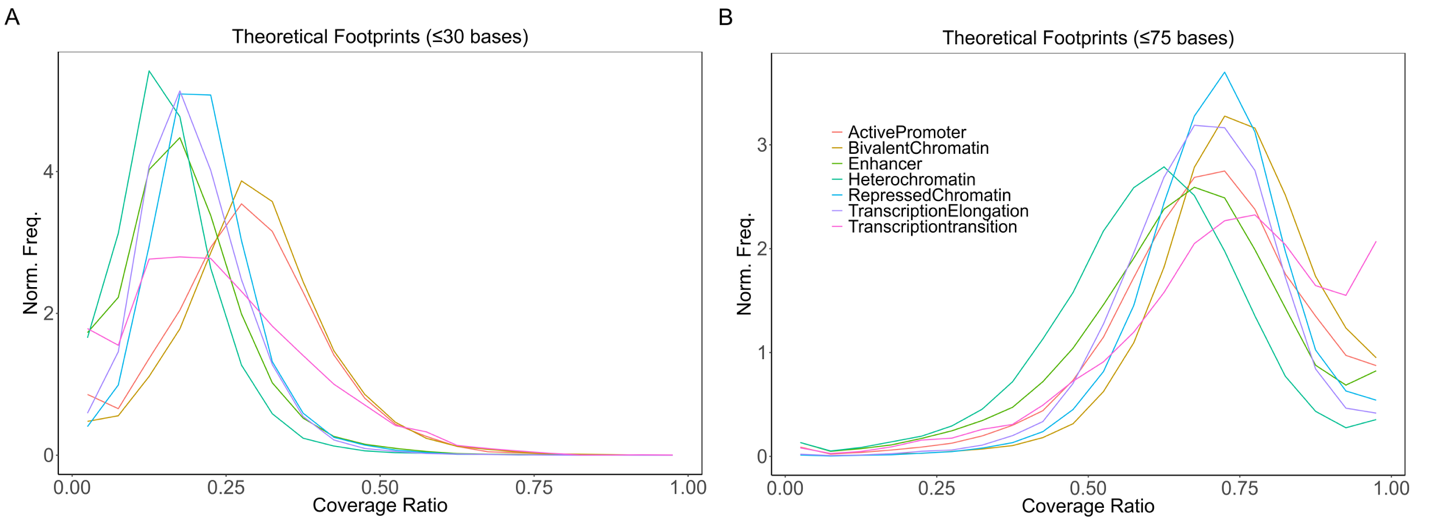** |
| --- |
| **Figure S5: Shortest theoretical footprint significantly overlaps with chromHMM-defined states.** Coverage ratio distributions for footprints ≤30 bp **(A)** and ≤75 bp **(B)** show a shift from low (~0.25) to high (~0.8) coverage with increasing footprint size, indicating greater overlap across chromatin states. Each curve represents a distinct chromatin state as indicated. ChromHMM data adapted from (Pintacuda et al., 2017). |

**Command-line usage of SMTrackR using a RESTful service**

We have also implemented a RESTful service for SMTrackR. Users can query for a heatmap, for example, using the following two commands (default parameters of the SMTrackR∷plotFootprints).

curl -JL "http://smtrackrest.iitr.ac.in:8000/api/generate?chromosome=chr2L&start=480290&stop=480320&label=peak229&format=json" -o peak229_response.json

curl -JL "http://smtrackrest.iitr.ac.in:8000/api/view/pdf/peak229" -o peak229.pdf

Values for acceptable parameters in SMTrackR∷plotFootprints function can be passed in the first command with “&” separator.

**References:**

Pintacuda, G., Wei, G., Roustan, C., Kirmizitas, B. A., Solcan, N., Cerase, A., Castello, A., Mohammed, S., Moindrot, B., Nesterova, T. B., & Brockdorff, N. (2017). hnRNPK Recruits PCGF3/5-PRC1 to the Xist RNA B-Repeat to Establish Polycomb-Mediated Chromosomal Silencing. *Molecular Cell*, *68*(5), 955-969.e10. https://doi.org/10.1016/j.molcel.2017.11.013
