# Supplementary figures and images for: SMTrackR: an R/Bioconductor package for mapping protein binding at individual DNA molecules
Source: Bioinform Adv. 2026 May 15;6(1):vbag091. doi: 10.1093/bioadv/vbag091 (PMC13287992; doi:10.1093/bioadv/vbag091)

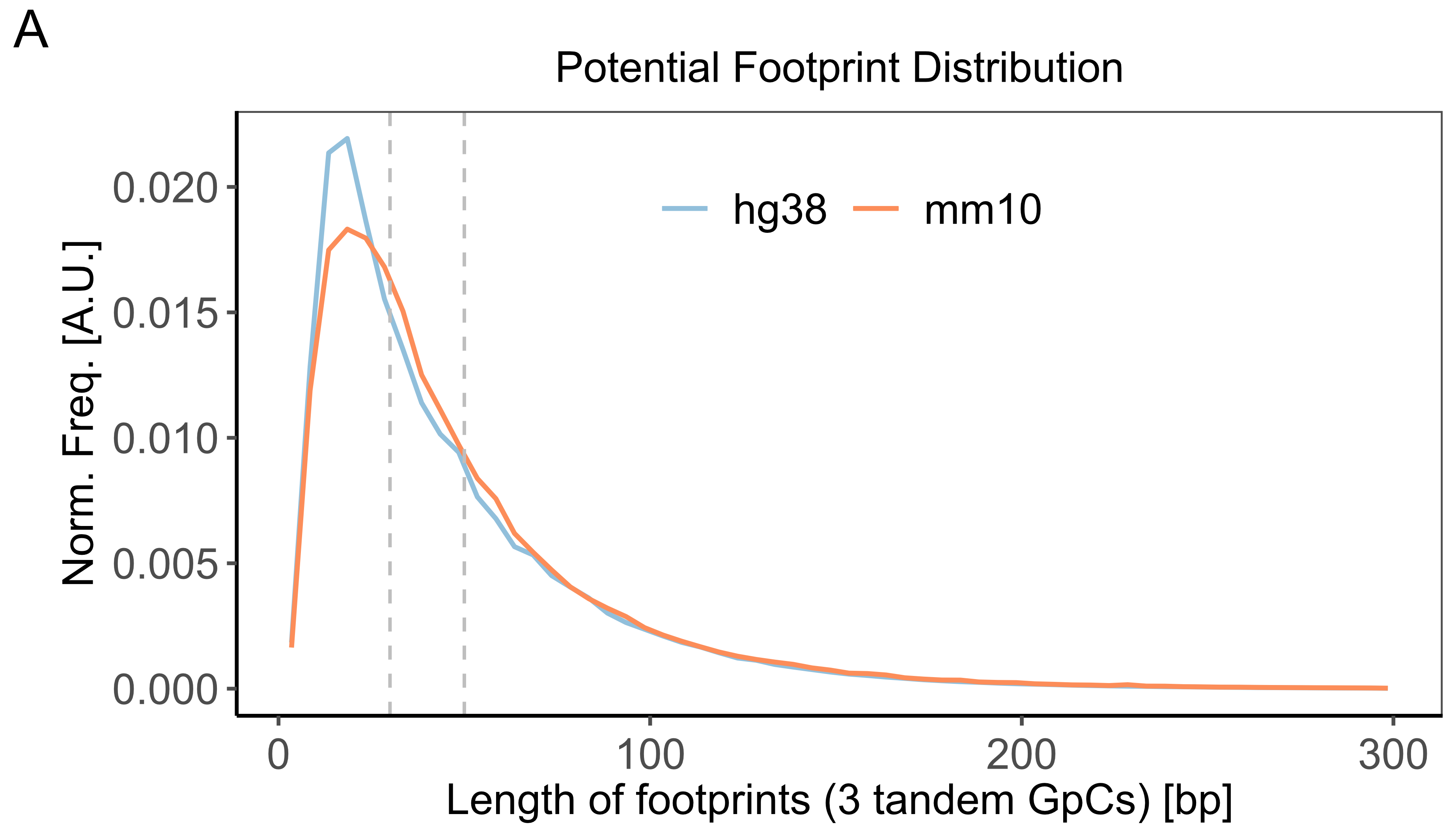

Supplement: vbag091_Supplementary_Data [file vbag091_supplementary_data.zip › figS4-600dpi.tiff]

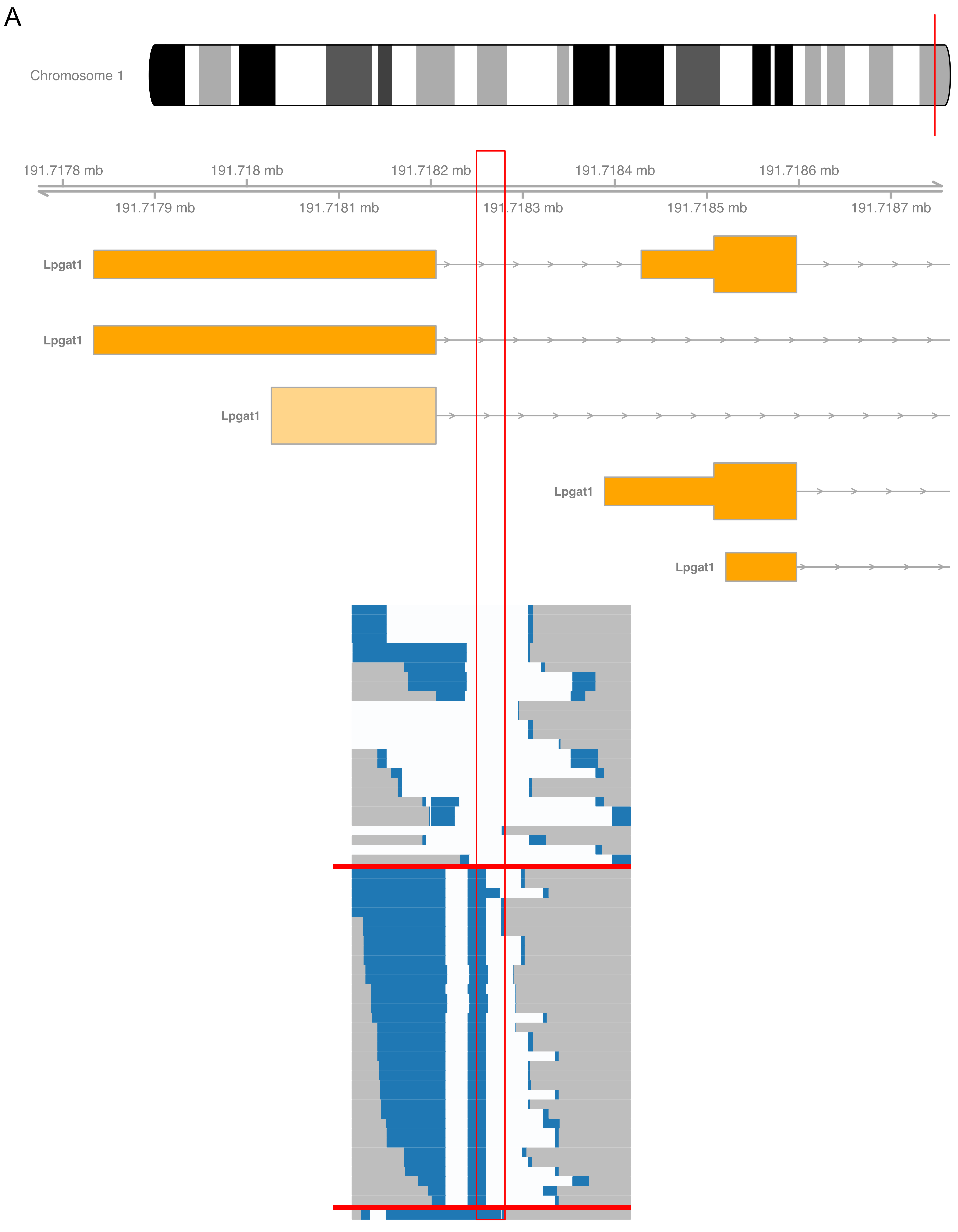

Supplement: vbag091_Supplementary_Data [file vbag091_supplementary_data.zip › figS3-600dpi.tiff]
